# Microscopic Polyangiitis following Silicone Exposure from Breast Implantation

**DOI:** 10.1155/2014/902089

**Published:** 2014-10-16

**Authors:** Judy Tan, Fuad Spath, Rakesh Malhotra, Zaher Hamadeh, Anjali Acharya

**Affiliations:** Department of Internal Medicine, Jacobi Medical Center, Albert Einstein College of Medicine, Bronx, NY 10461, USA

## Abstract

We describe a case of a patient who developed microscopic polyangiitis (MPA) in the setting of exposure to silicone after breast implantation. A 57-year-old Hispanic woman was admitted to our hospital with complaints of fever, cough, and hemoptysis. She had undergone silicone breast implantation two years prior to presentation. She was diagnosed as having microscopic polyangiitis (MPA) based on acute progressive renal failure, hematuria, pulmonary hemorrhage, and positivity for myeloperoxidase-anti-neutrophil cytoplasmic antibody (ANCA). A renal biopsy performed showed focal segmental necrotizing and crescentic glomerulonephritis. The patient received high dose steroids, cyclophosphamide, and plasmapheresis with remarkable clinical response. This case report raises the possibility of the development of MPA after silicone exposure from breast implantation.

## 1. Introduction

There have been a number of reported cases in which autoimmune syndromes have occurred following exposure to various chemicals. In 1964, Miyoshi et al. [1] first coined the term human adjuvant disease in their report of two patients who developed connective tissue-like disease after exposure to silicone-related substances during augmentation mammoplasty. Since this first observation, there have been numerous published cases relating silicone exposure to autoimmune rheumatic diseases. We, herein, report a patient who developed anti-neutrophil cytoplasmic antibody- (ANCA-) associated vasculitis following exposure to silicone from breast implantation.

## 2. Case Report

**Figure 1:**
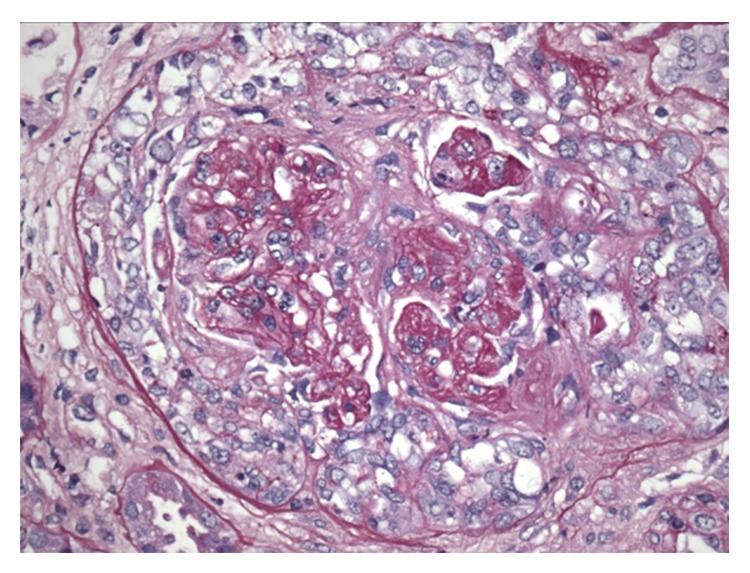
Glomerulus showing cellular crescent formation (H and E stain ×100).

A 57-year-old Hispanic woman presented to our institution with fever, cough, and hemoptysis. She had a history of type II diabetes mellitus, hypothyroidism, intermittent asthma, and nephrolithiasis. She also had left breast cancer for which she had a curative left mastectomy and a prophylactic right mastectomy with subsequent bilateral breast implantation with isotonic saline-filled silicone elastomer shell two years prior to presentation. Her physical examination was significant for bibasilar lung crackles. The rest of the examination was unremarkable. Radiologic imaging of her chest revealed left mid lung, basilar, and perihilar opacities. She was initially managed as a case of healthcare associated multifocal pneumonia with broad spectrum intravenous antibiotics. Because of her poor response to therapy and clinical deterioration, a bronchoscopy was pursued which revealed diffuse alveolar hemorrhage. Additional testing revealed microscopic hematuria (RBC: 44 per high-power field, elevated ESR and CRP, and antimyeloperoxidase antibody >100 U/mL (normal: <6 U/mL)) (Table 1). A preliminary diagnosis of microscopic polyangiitis (MPA) was made. A renal biopsy done revealed focal segmental necrotizing and crescentic glomerulonephritis, pauci-immune type (antimyeloperoxidase associated) with moderate activity and minimal chronicity, minimal tubular atrophy, and interstitial fibrosis (Figure 1). Immunofluorescence microscopy was negative for any significant immunoglobulins and complement deposition and no electron-dense deposition was detected by electron microscopy. She was treated with a combination of pulse dose of methylprednisolone, cyclophosphamide, and plasmapheresis with remarkable clinical response.

## 3. Discussion

Microscopic polyangiitis is an autoimmune disease characterized by a systemic vasculitis that predominantly affects the small blood vessels and is associated with the presence of anti-neutrophil cytoplasmic autoantibodies. Epidemiological evidence exists between silica exposure and ANCA associated diseases but the relationship remains disputed [2–7]. Iyoda et al. [2] report a similar case of microscopic polyangiitis after silicone breast implantation. Several published case-control studies demonstrate an association between ANCA-associated vasculitis and exposure to silica dust or other silica-containing compounds [8–10]. Gregorini et al. [8] estimated that patients with ANCA positive rapidly progressive glomerulonephritis (RPGN) were 14.0 times more likely to have been exposed to silica dust than their matched control subjects (95% confidence interval, 1.7 to 113.8; *P* < 0.001). Nuyts et al. [9] estimated that patients with Wegener's granulomatosis were 5.0 times more likely than age-, gender-, and region-matched control subjects to have been exposed to silica (95% confidence interval, 1.4 to 11.6). Hogan et al. [10] estimated that patients with ANCA-small vessel wall vasculitis were almost two times to be exposed to the highest score category for silica exposure compared with control subjects (OR 1.9; 95% CI 1.0 to 3.5; *P* = 0.05). Janowsky's study is a meta-analysis which studied nine cohort studies, nine case-control studies, and two cross-sectional studies failed to show association between silicone breast implants and development of connective tissue diseases [7]. Because of this discrepancy, it has been suspected that there might be a factor that predisposes certain people to developing autoimmune disease with exposure to silicone. This factor pertains to host susceptibility. Indeed, in a recent publication by Tsuchiya et al., an association of HLA-DRB1 ∗ 0901 with MPA and MPO-ANCA-positive vasculitis in Japanese patients has been reported [11].

The mechanism of silica exposure in the development of small vessel vasculitis is not well understood but several potential mechanisms have been proposed [12, 13]. One theory suggests that silica particles stimulate production of lymphocytes, including T cells and B cells, and that in certain clinical and genetic settings causes autoimmune disease as well as the production of autoantibodies, including ANCA [12]. A second theory suggests that silica particles activate monocytes and macrophages, resulting in the release of IL-1 or tumour necrosis factor-*α*, oxygen-derived free radicals, and lysosomal enzymes such as PR3 and MPO [13].

To our knowledge, this is the second report of MPA after exposure to silicone from breast implantation. An accumulation of such cases and further studies are necessary to clarify whether exposure to silicone or silicone-containing compounds or implants is related to the development of autoimmune disease.

## Figures and Tables

**Table 1:** Laboratory tests during hospital admission.

Laboratory test	Result
Hemoglobin (g/dL)	10.8
White blood cell count (×10^3^/mm^3^)	8.9
Serum creatinine (mg/dL)	1.3
ESR (mm/hr)	85
CRP (mg/dl)	22
Anti-myeloperoxidase antibody (U/ml)	>100

## References

[1] Miyoshi K., Miyaoka T., Kobayashi Y., Itakura T., Nishijo K. (1964). Hypergammaglobulinemia by prolonged adjuvanticity in man: disorders developed after augmentation mammoplasty. *The Japanese Medical Journal*.

[2] Iyoda M., Ito J., Nagai H., Sato K., Kuroki A., Shibata T., Kitazawa K., Sugisaki T. (2005). Microscopic polyangiitis after silicone breast implantation. *Clinical and Experimental Nephrology*.

[3] Chevallier A., Subra J. F., Renier G. (1991). Anti-myeloperoxidase antibodies and silicosis with renal involvement: new association or coincidental event. *American Journal of Kidney Diseases*.

[4] Talaszka A., Boulanger E., le Monies H. (1992). Silicosis, anti-myeloperoxidase antibodies and glomerular nephropathy. *Nephrologie*.

[5] Neyer U., Woss E., Neuweiler J. (1994). Wegener's granulomatosis associated with silicosis. *Nephrology Dialysis Transplantation*.

[6] Koeger A.-C., Lang T., Alcaix D. (1995). Silica-associated connective tissue disease: a study of 24 cases. *Medicine*.

[7] Janowsky E. C., Kupper L. L., Hulka B. S. (2000). Meta-analyses of the relation between silicone breast implants and the risk of connective-tissue diseases. *The New England Journal of Medicine*.

[8] Gregorini G., Ferioli A., Donato F., Tira P., Morassi L., Tardanico R., Lancini L., Maiorca R., Gross W. L. (1993). Association between silica exposure and necrotizing crescentic glomerulonephritis with P-ANCA and anti-MPO antibodies: a hospital-based case-control study. *ANCA-Associated Vasculitides: Immunological and Clinical Aspects*.

[9] Nuyts G. D., van Vlem E., de Vos A. (1995). Wegener granulomatosis is associated to exposure to silicon compounds: a case-control study. *Nephrology Dialysis Transplantation*.

[10] Hogan S. L., Cooper G. S., Savitz D. A., Nylander-French L. A., Parks C. G., Chin H., Jennette C. E., Lionaki S., Jennette J. C., Falk R. J. (2007). Association of silica exposure with anti-neutrophil cytoplasmic autoantibody small-vessel vasculitis: a population-based, case-control study. *Clinical Journal of the American Society of Nephrology*.

[11] Tsuchiya N., Kobayashi S., Kawasaki A., Kyogoku C., Arimura Y., Yoshida M., Tokunaga K., Hashimoto H. (2003). Genetic background of Japanese patients with antineutrophil cytoplasmic antibody-associated vasculitis: association of HLA-DRB1*0901 with microscopic polyangiitis. *Journal of Rheumatology*.

[12] Cohen Tervaert J. W., Stegeman C. A., Kallenbert C. G. M. (1998). Silicon exposure and vasculitis. *Current Opinion in Rheumatology*.

[13] Ueki A., Yamaguchi M., Ueki H. (1994). Polyclonal human T-cell activation by silicate in vitro. *Immunology*.

